# A meta-analysis of the impacts of internal migration on child health outcomes in China

**DOI:** 10.1186/s12889-016-2738-1

**Published:** 2016-01-22

**Authors:** Xiaoyue Sun, Mengtong Chen, Ko Ling Chan

**Affiliations:** 1 Department of Social Work and Social Administration, The University of Hong Kong, Pokfulam, Hong Kong; 2 School of Social Welfare, Yonsei University, Seoul, Republic of Korea

**Keywords:** Migrant children, Schooling, Physical health, Mental health, Child development

## Abstract

**Background:**

According to China’s 2010 population census, 38.81 million children migrated from rural to urban areas in Mainland China, a phenomenon that has attracted much scholarly attention. Due to the lack of quantitative synthesis of migrant children’s developmental outcomes, we undertook a meta-analysis to compare their developmental outcomes with those of their urban counterparts.

**Methods:**

We searched Applied Social Sciences Index and Abstracts (ASSIA), Australian Education Index, British Education Index, ERIC, ProQuest Education Journals, PsycINFO, Social Services Abstracts, Family & Society Studies Worldwide, Medline, Women’s Studies International databases and the Chinese CNKI database to identify relevant studies. Studies reporting physical and mental health outcomes of migrant children as well as potential protective and risk factors of child developmental outcomes were included. We assessed study quality using a quality assessment checklist.

**Results:**

We selected 25 studies from a total of 1592. Our results reveal that migrant children in public schools present significantly greater mental health problems and lower well-being than their urban counterparts, while migrant children in migrant schools do not present significantly different outcomes. In addition, migrant children were found to be more likely to be exposed to physical health risks due to limited utilization of health services. The disadvantageous health outcomes of migrant children were found to be related to a series of individual and social factors, including academic performance, social relationships, and discrimination.

**Conclusions:**

Migrant children are disadvantaged by the sociocultural circumstances in urban areas. Government should target them and provide appropriate support in order to improve their developmental status, which will have a positive impact on the stability and development of society.

## Background

Internal migration refers to human movement within a geopolitical unity, a phenomenon found in many regions such as Europe, the US, and China [1, 2]. Recently, migration has drawn attention from policymakers due to its magnitude and composition. A typical example is the EU, a supra-national entity comprising 28 member states. Unlike migration in the general sense, which imposes multiple conditions, like a visa, the EU places no restrictions on population movement across its internal borders. Thus, free movement between EU member states, though essentially an inter-state movement, can be more properly defined as “internal migration”. However, internal migration is not unique instance in EU, but has its counterpart. China, the biggest growing entity, comprises 34 provinces and sees a huge amount of labour migration. The government’s launch of reforms and the opening up policy in 1978 spurred large-scale migration. The introduction of a market economy made of China a large trading nation with a dynamic internal market and cheap labour. Huge waves of rural manpower flowed to urban areas which needed more labour for urban development. The number of internal migrants has increased continuously to the present.

Income and living standards affect the flow of population between EU member states. For instance, many people from Poland, Hungary, and Ireland move to Britain for better work and educational opportunities [3]. However, there is a scarcity of information about migrants in the EU [4]. Research on internal migration in China is more extensive compared to EU. In 2010, there were 221 million migrant workers in China, three-quarters of them rural–urban migrant workers. They are typically portrayed as poor and uneducated. Massive movement from rural to urban areas not only brings labourers out of the countryside, but also contributes to economic growth in destination areas. It is estimated that migrant workers have contributed 16 % of GDP growth in China in the last three decades, though the occupations open to migrant workers have been limited to specific sectors shunned by urban residents, for example, construction, domestic work, and cleaning work [5]. As more and more migrant workers obtained relatively stable jobs, they had their own families and many raised children in the cities. According to the 2000 census, 64 % of the heads of migrant families migrated with their spouses, and 61 % of migrant workers lived with their children in urban areas [6]. Migrant parents expect their children to be able to access better educational and developmental resources in urban areas so that they can improve their quality of life. However, the household registration system (*hukou*) still enforces restrictions on residential housing and income limiting rural–urban migrants’ access to education, housing, and health care [7]. The problems experienced by migrant families have drawn much attention as they have a huge impact on China’s social stability and social policies. This paper will therefore focus on internal migration after 1979, since it is only after that point that migration became a significant issue.

### Migrant children in international and Chinese contexts

The biggest concern for policy makers is that low-skilled workers are more likely than the average local individual to face health risks and less likely to utilize health services [8, 9]. Although most studies focus on adult migrants, the health and well-being of migrant children are very vulnerable to many threats. Migrant children may have greater difficulties with separation from family, disruption of education, and adaptation to new environments, and they are at greatest risk of having limited access to justice, education, and health care [10]. Migrant children present higher rates of underachievement and early drop-out at school, and also demonstrate more health problems than urban counterparts [11, 12]. Migrant children’s disadvantageous outcomes are mainly related to social and economic issues, for instance, service provision in host countries, socioeconomic status, age, and lack of social support [11, 13]. Notwithstanding, migration on such a large scale poses many challenges to social and economic development of China.

Internal migrant children in China have been also studied more extensively than migrant children in the EU. By 2010, there were about 36 million migrant children living with their parent(s) in urban areas, which indicates that one in eight Chinese children was a migrant [6]. In many migration destinations, such as Shanghai and Zhejiang, almost 50 % of children were from migrant families by 2010 [6]. However, local government restricted the provision of public services to people with local *hukou* only, which rules out all rural–urban migrants and their children. This poses challenges for current policies and social structure, which have a significant effect on social stability. In addition, as in the EU, migrant children in China experience stress caused by separation from family, disruption of education, and difficulties in adjusting to new social, cultural, and economic circumstances. Therefore, China can be considered a good laboratory for research on migrant children. Studying Chinese migrant children and families will improve our understanding of the global impacts of migration. A final important point is that migrant children contribute to the birth rate of their destination, and will be potential sources of labour in the near future. Therefore, policies and reforms are necessary to protect the development of migrant children, which will have implications in the global context.

Rural–urban migrant children have limited access to public services, such as education and health care, due to lack of urban *hukou*. Take school attendance as an example: in China, every citizen must complete 9 years of schooling, including 6 years of primary school education and 3 years of junior high school education. Children living in a place where their hukou registered are guaranteed to attend publically funded schools (public school) for the 9-year compulsory education, which is supported by local government. However, the right to accessing public education cannot mobile as people migrate because the financial support does not transfer between local governments. Regulations stipulate that public schools of host areas act as the main providers of compulsory education for migrant children; however, local government has not made on-the-ground improvements. Public schools set higher fees or entrance exams for migrant children, thereby denying them access to quality education [14]. Migrant schools specifically refer to schools open for children of migrant workers, privately run by migrant workers and charging lower fees. They were established as self-help mechanisms to meet the educational demands of migrant children. Social media and scholars are concerned about the poor sanitation, poor infrastructure, unqualified teachers, and unprofessional management of migrant schools; furthermore, migrant children are often criticized for academic failure and psychological and behavioural problems. Lack of access to public services not only affects migrant children’s school experience, it also puts their health and development at risk [15, 16]. On the one hand, poor living and learning conditions are harmful for child development. On the other hand, migrant children cannot access appropriate health education and prevention programmes in migrant schools. Public schools provide health education courses and regular examination services to students, thus delivering appropriate health education, information, and prevention. Migrant children are therefore at higher risk of health problems due to substandard living and school conditions.

Children are a very vulnerable population in the migration process. Evidence from other countries proves that migrant children lack legal, socioeconomic, and financial status [17]. There are a handful of literature reviews and systematic reviews of migrant children’s development in the international context. According to a literature reviews conducted by Chan and colleagues [17], children migrating to western countries meet with cultural and language barriers and limited health care services. For instance, most children migrating from less-developed countries to developed countries face problems like incomplete immunization and poor nutrition, physical violence, psychological depression, and symptoms of post-traumatic stress disorder (PTSD) [18–20]. Further, children are highly likely to internalize that psychological depression and express it in problematic behaviours [21, 22]. Therefore, migrant children experiencing physical and social stressors are more likely to have mental health problems.

However, few comprehensive reviews have been conducted on the physical and mental health outcomes of migrant children in Mainland China. According to a review conducted by Zhang [23], Chinese scholars mainly used specialized mental health instruments to measure the psychological well-being of migrant children. Some scholars also use single item like social adaptation and loneliness. As reported in a systematic review conducted by Mou [24] and his colleagues, scholars have compared test results between migrant children and urban children and generally found that migrant children present higher rates of psychological disorder symptoms. However, this systematic review has two limitations. First, it fails to take a neutral standpoint, only including studies showing that migrant children were disadvantaged in health outcomes. Second, it fails to point out the heterogeneity of migrant children, that is migrant children in migrant children and those in public school are not identical. In addition, researchers have identified correlates of children’s psychological well-being from the level of family, to school and society, for instance, family resources, parenting, migration, interpersonal relationships, *hukou*, and distribution of public resources [25, 26]. Scholars have acknowledged that the factors mentioned above influence not only the mental health of migrant children, but also their physical health and educational outcomes. However, there is a lack of statistical synthesis of migrant children’s developmental outcomes and their relationships with individual and social factors.

In this study, we conducted a meta-analysis of the effects of parental migration on child development in studies conducted between 1979 and 2015. We address four major questions: 1) Are migrant children at higher risk of physical health problems compared to their urban counterparts? 2) Do migrant children have more mental health problems than their urban counterparts? 3) Does public school attendance play a protective role for the well-being of migrant children? 4) What are the protective/risk factors of mental health outcomes for all migrant children regardless of the school type?

## Methods

### Search strategy

We searched Applied Social Sciences Index and Abstracts (ASSIA), Australian Education Index, British Education Index, ERIC, ProQuest Education Journals, PsycINFO, Social Services Abstracts, Family & Society Studies Worldwide, Medline, Women’s Studies International databases and the Chinese CNKI database to identify relevant studies published in English and Chinese between 1979 and 2015. For grey literature, we manually searched the references of review articles, CNKI, and Google Scholar and also contacted authors of published articles. We used the search terms “migrant children”, “left-behind children”, and “China” (Table 1). We only included studies published in English and Chinese. Two reviewers retrieved and independently screened full-text copies of articles based on four inclusion criteria: 1) studies must be a cross-sectional, case control, or panel study; 2) studies must be quantitative with a sample equal to or larger than 30; 3) studies must focus on the health outcomes of school-age children who are affected by parental migration; and 4) studies must report either rates or raw data for health problems or risks to enable their calculation.

**Table 1:** Search terms used for English-language and Chinese literature

Group 1	Group 2	Group 3
Migrate/Migrant/ Floating/Left behind/Migration	Child/Student/ Teen/Adolescent	China Chinese

We excluded studies that matched four exclusion criteria: 1) studies about international migration of Chinese population (e.g. Chinese immigrants in the US); 2) studies only reporting on the left-behind children of migrant workers without providing information on migrant children; 3) studies with insufficient data (e.g. sample size, standard difference).

### Quality assessment and data extraction

Two reviewers independently assessed the quality of all identified articles based on a quality assessment checklist and given a grade. The checklist, shown in the Appendix, comprises seven items regarding reporting and methodological quality. The scoring method involved dividing the total score by the number of applicable items, and then one of three grades was assigned to studies accordingly: 0–33 % represents bad quality, 33–66 % is satisfactory, and 67–100 % is good quality. Table 2 reports the scores; the highest was 100 %, which means that this particular article satisfied all criteria. Most studies used probability sampling; some reported detailed sampling procedures while others did not. The methodological quality varied significantly across selected studies. Most studies employed cross-sectional design except for three longitudinal studies. Only two studies recruited migrant children from both migrant and public schools, enabling within-group comparison [27, 28]. The other studies only recruited migrant children from one type of school. However, all of the 25 studies were eligible for inclusion because they provided sufficient data to compute effect sizes and employed satisfactory methodology (e.g., response rate, sample size, and outcome measurements). Inter-rater reliability was computed using Cohen’s kappa, and we obtained a high level of agreement (weighted κ = 0.91).

**Table 2:** Study characteristics

Study	Study characteristics						Health outcomes		
	Method score	Study design	No. of participants (MC/LC)	Sub-groups	Protective factors	Risk factors	PH	Positive mental health	Negative mental health
Zhou (2006) [28]	6	CS	971/164	Migrant children in ms/ps	TS	–	–	–	LO/DE
Lu et al. (2008) [30]	5	LS	960/947	–	–	–	HS	–	–
Zou et al. (2008) [42]	5	CS	1018/447	–	AP/TS	–	–	AD	IP/EP
Li et al. (2009) [37]	4	CS	1146/-	–	TS	–	–	AD	IP/EP
Chen et al. (2009) [35]	6	CS	411/518	Migrant children in ms	AP	–	–	PO	DE
Liu & Shen (2009) [51]	5	CS	1298/287	–	–	DI	–	–	IP
Wong et al. (2009) [48]	7	CS	625/-	–	PC/PS/TS	DI	–	–	IP
Lin et al. (2009) [27]	5	CS	1164/525	Migrant children in ms/ps	–	DI	–	–	AN/ DE/ LO
Gao et al. (2010)	4	CS	91/47	–	–	–	OH	–	–
Wong et al. (2010) [46]	7	CS	625/-	–	PC/PS/TS	DI	–	AD	IP
Zeng (2010) [40]	5	CS	684/545						IP/EP
Au et al. (2011) [43]	4	CS	188/97	–	PC/PS	–	–	PO	–
Fan & Chen (2012) [49]	5	CS	558/-	–	–	DI	–	–	DE
Fan et al. (2012) [50]	6	CS	1164/-	Mixed	–	DI	–	AD	–
Ji et al. (2012) [34]	3	CS	10,277	–	–	–	NU/GH	–	–
Mao et al. (2012) [38]	6	CS	484/128	Migrant children in ps	PC	–	–	AD	–
Wu et al. (2012) [47]	6	CS	806/-	Mixed	–	–	–	PO	–
Liu et al. (2013) [52]	3	CS	1551/-	Mixed		DI		AD	–
Lu & Zhou (2013) [41]	7	LS	1259/-	Mixed	AP	DI	–	–	LO
Yuan et al. (2013) [39]	6	LS	1164/-	Migrant children in ps	–	–	–	AD	–
Chen (2014) [56]	4	CS	4256/112,340	–	–	–	NU/GE	–	–
Cheung (2014) [44]	7	CS	482/838	Mixed	PC/PS	–	–	PO	–
Fang et al. (2014) [45]	4	CS	301/-	–	PC/TS/PS	–	–	AD	–
Gao et al. (2015) [36]	6	CS	1019/447	Migrant children in ms/ps	–	–	–	–	IP/EP
Wu & Luo (2015) [31]	4	CS	198/202	–	–	–	HS	–	–

Notes: *CS* cross sectional study, *LS* longitudinal study, *ST* school type, *AP* academic performance, *TS* teacher-student relationship, *PC* parent–child relationship, *PS* peer support, *HS* health services, *HP* health problems, *PH* physical health, *IP* internalizing problems, *EP* externalizing problems, *PO* positive mental outcomes, *OH* oral health, *GH* general health, *NU* nutrition, *LO* loneliness, *DE* depression, *AN* anxiety, *DI* discrimination, *ms* migrant school, *ps* public school

### Outcome measures

The key outcomes of interest are the physical and mental health outcomes of migrant children in urban areas. The measurements of mental health outcomes include both positive and negative outcomes, which are analysed separately. Although different studies employed different instruments, the measure constructs were similar enough for data synthesis [29]. Positive mental outcomes include emotional well-being and sociocultural adjustment; negative mental outcomes include internalizing and externalizing problems such as loneliness, depression, and anxiety, etc. Physical health outcomes include uninsured rate, unvaccinated rate, oral health problems, and other general health problems (anaemia, diarrhoea, malnutrition, etc.). We grouped outcomes related to utilization of health services (vaccination and insurance) and health problems in two sets, and calculated odds ratios (OR) to compare the health risks of migrant children with those of local children. These outcomes were then pooled together as overall health risk outcomes.

### Data analysis

We summarized the selected studies by descriptive analysis; the main characteristics were tabulated, including publication information (e.g., year and author), methodological characteristics (e.g., methodology grade, sample size, moderators, etc.), and outcome measurements (e.g., positive and negative mental outcomes). We calculated the effect size of mental health outcomes with standard difference in means with 95 % CI of the group of migrant children and local children in cities. In order to test the effects of the moderator variables, we grouped the studies according to available study characteristics (outcome type). We also calculated the odds ratios (OR) with 95 % CIs for the risk of physical health problems among migrant children. Random effects models were employed to combine studies. We used the Q statistic to estimate heterogeneity. We calculated effect size and conducted heterogeneity tests and moderator analyses using the Comprehensive Meta-Analysis software (3^rd^ version).

## Results

### Study characteristics and participants

**Fig. 1:**
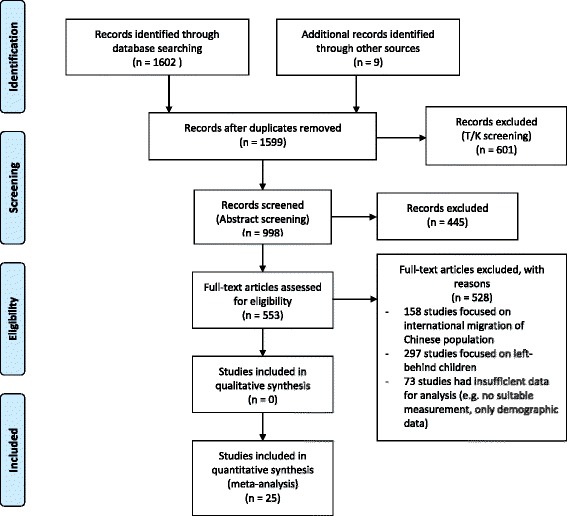
Screening process of English-language and Chinese resources

We identified 673 studies from the English databases and 12 were eligible for inclusion. We obtained 917 studies from the Chinese database and 12 were included for analysis. We identified nine grey literature, whilst only one was eligible for analysis. In total, 25 studies were included for analysis (Fig. 1). Table 2 summarizes the characteristics of the 25 studies. Twenty provided data for mental health outcomes, far fewer for physical health outcomes. Among the mental health studies, eight focused on examining the mental health of migrant children, including migrant children in migrant and public schools. Measures of mental health included both negative and positive outcomes, in terms of internalizing problems (depression, anxiety, loneliness, etc.), externalizing problems, and adaptation. Twelve studies reported individual and social correlates of mental health outcomes, including academic performance, social support, and discrimination. Five studies reported physical health outcomes of migrant children, including rates of health service coverage and prevalence of health problems.

The sample sizes across the selected studies ranged from 138 to 116,596. In total the selected studies used 150,232 participants, representing a large sample. The participants were recruited from different sources, such as migrant schools, public schools, and communities. Most migrant children were from low-income families. Though the school age in China is six to 15, participants in mental health studies were roughly between 9 and 19 years old. The youngest participants in the physical health studies were 5 years old.

#### Physical health outcomes

Only five studies focused on the physical health outcomes of migrant children. Two studies reported children’s health service coverage and utilization, including health insurance and the EIP vaccine [30, 31]. Three studies examined the prevalence of child health problems.

**Fig. 2:**
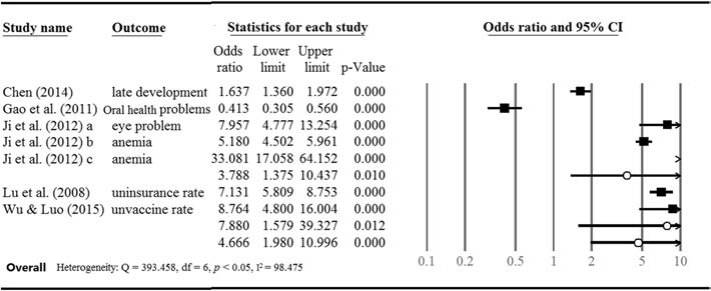
Physical health risks of migrant children (cf. local children in cities)

Measurements used included dental examination, nutrition examination, and other general health examinations [32–34]. Figure 2 shows the random effect sizes pooled by all the outcomes of the selected studies. First, we pooled the data in order to calculate the overall odds ratio of health risks, comparing the prevalence of the problems of migrant children and local children who reside in the region in which their household is registered. Pooling of the prevalence of lack of health services and health problems enables calculation of the health risks of migrant children for comparison with local children in cities. The statistics show higher health risks among migrant children (OR = 4.666, *p* < 0.05), so migrant children are exposed to much higher physical health risks than local children. Notwithstanding, a wide variance in the effect sizes of all the studies was observed; about 98 % of total variation is explained by heterogeneity among the studies (I^2^ = 98.74 %). Next, we coded the studies into two subgroups according to outcome type: lack of health service utilization and health problems. Migrant children presented a higher likelihood of having physical health problems than their urban counterparts (OR = 3.788, *p* < 0.05); they are also unlikely to use health insurance and have less EIP vaccine coverage than their urban counterparts (OR = 7.880, *p* < 0.05).

#### Mental health outcomes

**Fig. 3:**
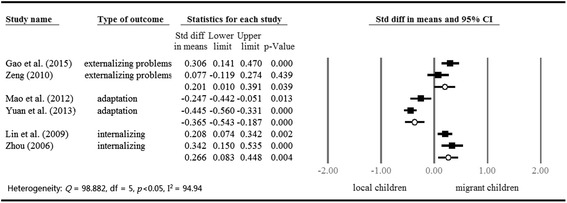
Mental health outcomes of migrant children in public schools (cf. local children in cities)

**Fig. 4:**
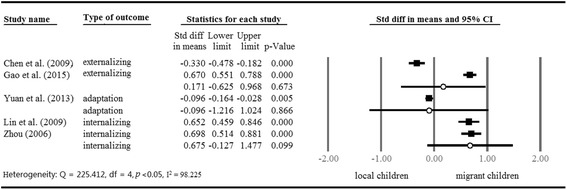
Mental health outcomes of migrant children in migrant schools (cf. local children in cities)

School age is a peak time of emerging psychosocial and behavioural problems, such as loneliness, depression, anxiety, smoking, and so on. Studies have reported that migrant children are more vulnerable to psychological problems than local urban children [27, 28, 35–37]. In addition, migrant children are disadvantaged in positive mental health outcomes, including life satisfaction and sociocultural adjustment [35, 38–40]. We pooled and compared the results of migrant children and local children according to different types of school and outcomes; the results are presented in Figs. 3 and 4. The mental health outcomes of migrant children in public schools were extracted from six studies; those of migrant children in migrant schools were extracted from five studies. Two studies reported outcomes of migrant children in both public and migrant schools with a comparison group composed of local children; however, one group can only contribute once in an analysis [27, 37]. Therefore, comparison between migrant and local children was conducted by migrant children’s school attendance type to avoid dependency. We also grouped studies according to the type of outcome due to the variation in measurements, including adaptation and internalizing and externalizing problems. Further, we examined the potential protective and risk factors of mental outcomes, including academic performance, parent–child relationship, peer relationship, teacher-student relationship, and perceived discrimination (Table 3).

**Table 3:** Protective and risk factors of health outcomes

Protective/Risk factors	Outcomes	No. of studies	Correlation	Heterogeneity			
				Q	df	*p*	I
Academic performance	Negative mental outcomes	2	−0.293 [−0357, −0.226]; *p* < 0.05	6.504	2	0.000	69.249
Parent–child relationship	Positive mental outcomes	5	0.141 [0.047, 0.233]; *p* < 0.05	18.079	4	0.000	77.875
	Negative mental outcomes	1	−0.019 [−0.233, 0.196]; *p* > 0.05	0	0	1	0
Peer support	Positive mental outcomes	4	0.106 [0.034, 0.177]; *p* < 0.05	0.468	3	0.926	0
	Negative mental outcomes	2	0.002 [−0.066, 0.071]; *p* > 0.05	1.971	1	0.160	49.264
Teacher-student relationship	Positive mental outcomes	6	0.241 [0.129, 0.347]; *p* < 0.05	35.743	4	0.000	88.81
	Negative mental outcomes	3	−0.125 [−0.175, −0.075]; *p* < 0.05	3.391	2	0.183	41.023
Discrimination	Positive mental outcomes	3	−0.338 [−0.462, −0.201]; *p* < 0.05	4.774	2	0.000	58.104
	Negative mental outcomes	5	0.255 [0.138, 0.365]; *p* < 0.05	36.381	4	0.000	89.005

We used standard difference in means to calculate the differences between migrant and local children in terms of mental health outcomes. In the comparison between migrant children in public schools and local children, we observed a wide variance in the effect sizes of studies about children’s mental outcomes (Q = 98.882, I^2^ = 94.94). Figure 3 shows that migrant children in public schools scored higher in externalizing problems (SDM = 0.201, *p* < 0.05) and internalizing problems (SDM = 0.266, *p* < 0.05), and scored lower in positive mental outcomes than local children (SDM = −0.365, *p* < 0.05). However, Fig. 4 shows that there was no significant difference between migrant children in migrant schools and local children in terms of mental health outcomes.

### Protective and risk factors of health outcomes

Children’s psychosocial development is closely related to individual, household, social, and other contextual factors; researchers attribute the differences between migrant and urban children to academic performance, social support, and discrimination in particular. Table 3 shows the protective and risk factors of mental health outcomes, including academic performance, parent–child relationship, peer support, teacher-student relationship, and discrimination. Two studies reported that better academic performance is related to better mental health outcomes [41, 42], five studies reported that a better parent–child relationship is related to better mental health outcomes and fewer mental health problems [43–47]; four studies reported that stronger peer support is related to better mental health outcomes and fewer mental health problems [43–46]; six studies reported that a better teacher-student relationship is related to better mental health outcomes and fewer mental health problems [37, 42, 44–46, 48]. In addition, eight studies reported that perceived discrimination is related to increased mental health problems and decreased mental well-being [27, 41, 46, 48–52]. Therefore, the academic performance of individuals and their social relations to parents, peers, and teachers play a significant protective role in mental health outcomes. Discrimination is a risk factor of child mental health which demonstrates a negative and moderate correlation with mental well-being (Corr = −0.338, *p* < 0.05).

## Discussion

Our results show migrant children are exposed to higher physical health risks than local children. The results show that migrant children are less likely to use health services, which are usually delivered free to local children but not to migrant children. The utilization of health insurance and EIP vaccination is dependent on parental decisions, which are mostly constrained by financial ability and *hukou* restriction on their access to urban services. Many migrant children had problems with being insured and reimbursement application when they were not living in their *hukou* registration place, so the health care system should adapt to solve the problems of frequent mobility and remote application for reimbursement. In the meanwhile, the government should provide fiscal incentives for local government in order to promote the health care of migrant children. The local government can target migrant children and involve them in urban health insurance system with lower payment standard in order to reduce financial burden on migrant families.

With regard to the mental health problems, migrant children in public schools have more internalizing and externalizing problems than local urban children. In addition, migrant children are more likely to have problems with adaptation to school, social, and cultural circumstances in urban areas. Although the standard difference in the means of migrant children in migrant schools and local children is not statistically significant, migrant children in migrant schools have previously been found to score highest in loneliness, depression, and anxiety scales, followed by migrant children in public schools, whilst local children scored the lowest [37]. The current study did not find that trend, which provides evidence countering stereotypes of migrant children. However, we expect that migrant children would benefit from more public resources in public schools, which would give them more opportunities to integrate into the mainstream.

In addition, exploration of protective and risk factors explained the potential stressors that threaten the mental well-being of migrant children. Take academic performance and social relations for instance: Chinese schools pay too much attention to children’s academic performance while neglecting their social and emotional functioning. Many studies have found that migrant children perform worse in school than their local classmates, so they are more likely to be looked down on and to feel more negative emotions [15, 53–55]. There is evidence that the parents of migrant children are unlikely to employ appropriate parenting skills and provide a supportive environment, and migrant children have been found to perceive less parental warmth and support than urban children [46]. The quality of migrant children’s relationships with parents, peers, and teachers may be low, causing them to feel lonely and depressed, and these factors may affect their psychological development [56]. It is difficult for migrant children to integrate into urban society when they feel uncomfortable with their social relationships, norms, and school. Thus, teachers and school managers should pay attention to changes of school climate and strengthen social relationships in order to protect the mental well-being of migrant children and reduce the stresses and difficulties they face.

This study indicates that rural–urban migrant children face similar embarrassing situations to migrant children in western societies. Rural–urban migrant children may feel uncomfortable with language, social connections with local children, and urban norms, which also frustrate international migrant children. The current study thus sheds further light on the health and well-being of migrant children in the global context.

### Strengths and limitations

The current study has synthesized the health outcomes of migrant children in China. It took a neutral standpoint that pooled positive and negative outcomes of health and well-being. We focused on comparison of migrant and local children, measuring a range of outcomes. Confirming the disadvantaged status of migrant children in terms of physical and mental health, we further explored the potential protective and risk factors of health outcomes. The results contribute to the current understanding of the developmental circumstances of migrant children in urban areas, especially the discovery that migrant children share some common outcomes with their urban counterparts.

However, the current meta-analytical study has several limitations. First, meta-analysis relies on the information available that reported by other studies, but the information extracted from relevant studies was limited. For instance, many studies failed to recruit migrant children from both migrant and public schools, whilst some did not indicate the sample sizes of subgroups (e.g. migrant children in migrant schools and in public schools), which made it impossible to compute the prevalence of health problems for these subgroups. Another problem with limited information is that we cannot control for age in our analysis; some studies provided the average age of participants, while some provided the range of participants’ age. It may influence the accuracy of the effect size without controlling for age. Second, some studies only reported limited outcomes, so there was insufficient data to test potential protective and risk factors of the physical health outcomes of migrant children. For instance, we could not test the relationship between socioeconomic status and health outcomes. Third, though the study followed a strict search strategy and quality assessment, we only included one eligible grey literature. This limits our control of publication bias, which may affect the generalization of study results.

## Conclusions

The current meta-analysis sheds light on the profound consequences of internal migration on some child outcomes. We find that there is little research on the victimization of migrant children, which made it impossible to conduct analysis of that topic. However, according to news reports, the victimization of migrant children has been a frequent occurrence in recent years. Parents of migrant children tend to work longer hours and pay less attention to care giving, so migrant children are more likely to be neglected and victimized when alone. This topic deserves scholarly research to enrich our understanding of the developmental and well-being status of migrant children.

## Appendix

### Quality assessment checklist

1. Did the study report sampling procedure?

**Table:**

a. No	0
b. Yes	1

2. Did the study define the outcome as psychological/physical well-being or problems?

**Table:**

a. No	0
b. Yes	1

3. Was the measurement of mental health/physical health outcomes objective?

**Table:**

a. No	0
b. Yes	1
c. Not applicable	NA

4. Was the response rate of the study over 60 %?

**Table:**

a. No	0
b. Yes	1

5. Did the study report outcomes of migrant children from both migrant and public schools?

**Table:**

a. No (migrant children of one type of school or not mentioned)	0
b. Yes	1
c. Not applicable	NA

6.Did the study adjust for confounding factors?

**Table:**

a. No	0
b. Yes	1
c. Not applicable	NA

7. Were the migrant children and local urban children recruited from the same population?

**Table:**

a. No	0
b. Yes	1
c. Not applicable	NA
